# Baseline estimated pulse wave velocity (ePWV) and its longitudinal change are associated with incident frailty in older Chinese adults: a prospective cohort study

**DOI:** 10.3389/fpubh.2026.1926256

**Published:** 2026-10-28

**Authors:** Yu Bao, Dongmei Yu, Yue Wei, Junxiu He, Dongyi Feng, Yujin Li, Yujuan Zou, Haifan Zhao, Hong Cheng, Haiqing Cai, Xing Chen, Fei Wang, Yan-Feng Zhou, Xiaobo Yang

**Affiliations:** 1 Department of Occupational Health and Environmental Health, School of Public Health, Guangxi Medical University, Nanning, Guangxi, China; 2 Department of Social Medicine, School of Public Health, Guangxi Medical University, Nanning, Guangxi, China; 3 School of Public Health and Health Management, Guangxi Health Science College, Nanning, China

**Keywords:** arterial stiffness, estimated pulse wave velocity, frailty, longitudinal change, older adults

## Abstract

This study aimed to examine the longitudinal associations of estimated pulse wave velocity (ePWV) and its longitudinal change with incident frailty in older adults. A total of 2,016 older adults (≥60 years, baseline non-frail) were included in baseline analysis, selected from an initial cohort of 4,129. Of these, 1,137 had repeated ePWV data for change analysis. Arterial stiffness was measured using ePWV, and its longitudinal change from baseline to follow-up was defined as ΔePWV. Frailty was assessed using a frailty index (FI), analyzed as both continuous and binary outcome (FI ≥ 0.25). Multivariable regression models evaluated associations of baseline ePWV and ΔePWV with incident frailty. After a mean follow-up of 7 years, 394 of 2,016 participants (19.5%) developed frailty. Each 1 m/s increase in baseline ePWV was associated with higher frailty risk (OR = 1.21, 95% CI: 1.10–1.33); the highest quartile had more than twice the risk of the lowest (OR = 2.11, 95% CI: 1.36–3.28). Among 1,137 participants with repeated measures, each 1 m/s increase in ΔePWV was associated with 21.4% higher frailty odds (OR = 1.21, 95% CI: 1.03–1.43), and rapid increase group had higher risk than decline/stable group (OR = 1.91, 95% CI: 1.13–3.23). Results were consistent in continuous FI and most sensitivity analyses. However, after excluding hypertension and cardiovascular disease from the FI, the associations were attenuated and no longer statistically significant. These findings indicate that higher baseline ePWV and increases in ePWV over follow-up were associated with an increased risk of incident frailty, although part of this association may be related to the cardiovascular components within the FI. ePWV may serve as a useful indicator for identifying individuals at elevated risk of frailty.

## 1 Introduction

Frailty, a geriatric syndrome common in older adults, involves physiological deterioration across multiple systems (1). This condition contributes to elevated risks of disability (2), hospitalization (3), and mortality (4), posing a growing public health challenge in aging societies. Identifying modifiable risk factors is crucial for implementing effective prevention strategies.

Arterial stiffness, a central marker of vascular aging, has emerged as a potential contributor to systemic functional decline (5–7). Although carotid-femoral pulse wave velocity (cfPWV) is the gold standard (5, 8), its measurement complexity limits widespread clinical use. Estimated pulse wave velocity (ePWV) is a surrogate metric calculated from age and mean blood pressure via a validated formula, and has been shown to have predictive value comparable to directly measured cfPWV (9). ePWV requires no direct arterial waveform measurement. Its simplicity and reproducibility make it suitable for repeated assessments in large longitudinal cohorts, where directly measured PWV may not be feasible. Nevertheless, as an estimate, ePWV cannot fully capture the complexity of arterial wall properties. Current evidence linking directly measured arterial stiffness to frailty is largely derived from cross-sectional studies (10–12). Several recent longitudinal studies have investigated the associations of cfPWV and baPWV with frailty (13, 14). However, the longitudinal association between ePWV, a more readily accessible surrogate marker of arterial stiffness, and incident frailty remains poorly defined. Furthermore, arterial stiffness is not static but a dynamic process (15), whether changes in ePWV independently contribute to subsequent frailty risk has not been established.

To fill these research gaps, we performed a prospective cohort study aimed at systematically answering two core questions: (1) whether baseline ePWV is associated with incident frailty, and (2) whether longitudinal changes in ePWV are associated with subsequent frailty risk. By clarifying these relationships, this study aims to provide longitudinal evidence on the role of vascular aging in frailty development and offer a scientific basis for assessing frailty risk using a simple metric.

## 2 Methods

### 2.1 Study design and participants

Data for this community-based prospective cohort study were derived from a survey of older adults residing in the Hongshui River Basin, Guangxi, China. The baseline survey was carried out during 2016–2018, enrolling a total of 4,621 permanent residents aged ≥60 years. Details of the recruitment procedures have been reported previously (16).

For the present analysis, participants were selected as follows (Figure 1). We first excluded individuals with incomplete baseline data (defined as >10% missing variables (17) used to construct the frailty index (FI), *n* = 61), those who were frail at baseline (*n* = 428), and with missing baseline blood pressure data (*n* = 3). This left 4,129 non-frail participants. Of these, 757 died before follow-up, 1,034 were lost to follow-up, and 322 had insufficient follow-up frailty data. Consequently, 2,016 participants were included in the primary analysis to examine the prospective association between baseline ePWV and frailty. The secondary analysis of changes in ePWV was restricted to 1,137 participants with both baseline and follow-up ePWV measurements. Blood pressure was measured by trained research staff using a standardized protocol at both baseline (2016–2018) and follow-up (2024), with the same measurement protocol applied at both time points.

**Figure 1:**
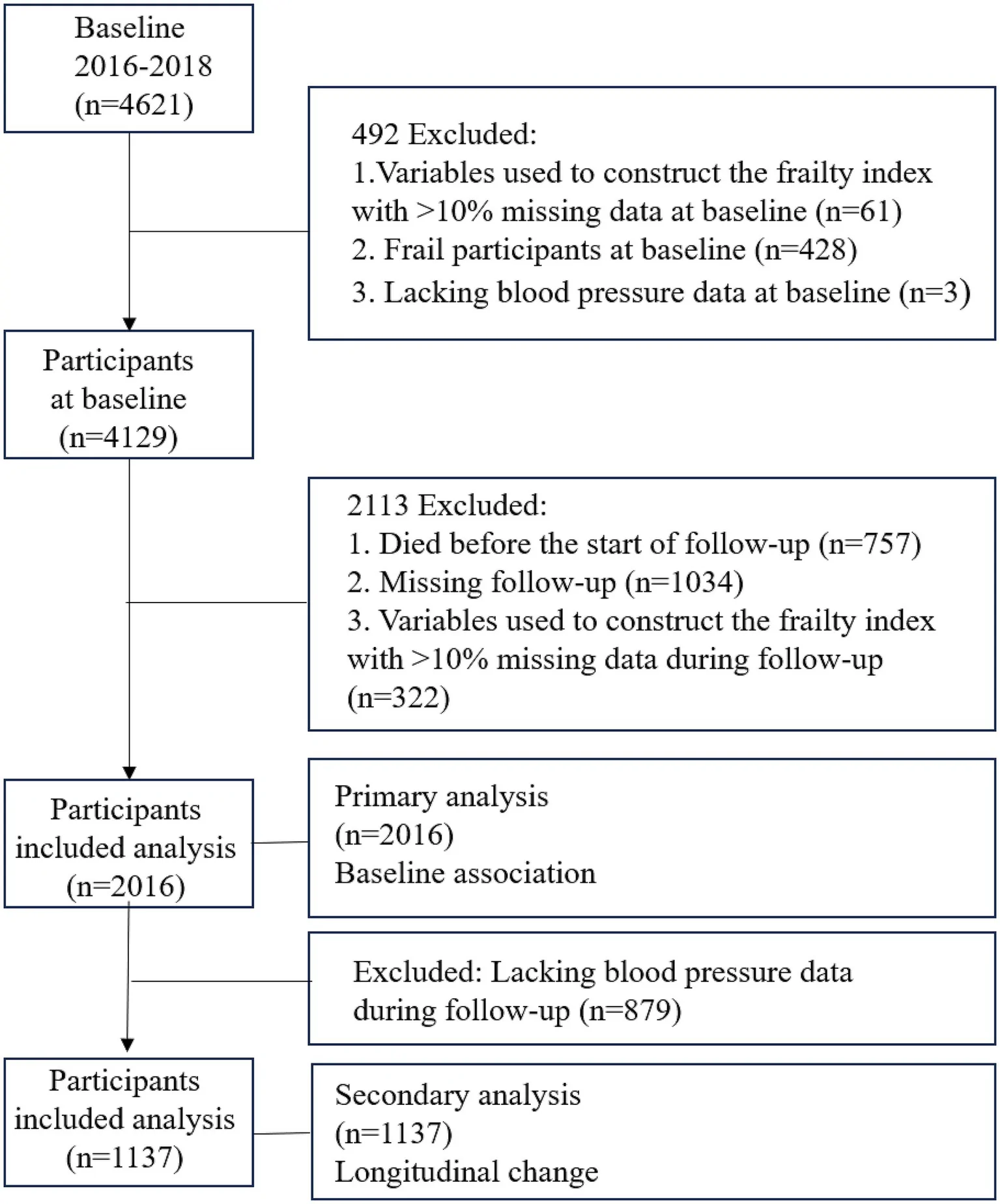
Flow diagram of the study participants.

### 2.2 Assessment of arterial stiffness

Arterial stiffness was assessed using ePWV. The ePWV values were obtained using a validated formula (9, 18) that incorporates age and mean blood pressure (MBP): ePWV = 9.587–0.402 × Age + 0.004560 × Age^2^–0.00002621 × Age^2^ × MBP + 0.003176 × Age × MBP − 0.01832 × MBP. Here, MBP = DBP + 0.4 × (SBP – DBP), where SBP and DBP denote systolic and diastolic blood pressure.

The analytical variables included baseline ePWV and its longitudinal change (ΔePWV). This change quantified as the difference between follow-up and baseline values. Because no established clinical threshold exists for ΔePWV, participants were categorized according to its distribution. Using the 25th (0.920 m/s) and 75th (2.261 m/s) percentiles as cutoffs, participants were classified into three groups: decline/stable (<P25), slow increase (P25–P75), and rapid increase (>P75) (19, 20).

### 2.3 Assessment of frailty

Frailty was quantified using a FI constructed according to standard procedures (21). Briefly, 32 health deficit items were selected from four domains: chronic diseases, physical function, general health status, and symptoms/signs (Supplementary Table S1). Deficit severity was graded from 0 (completely healthy) to 1 (fully impaired). The FI was calculated as the sum of available item scores divided by the total number of items. For participants with ≤10% missing items, the denominator was adjusted to the count of non-missing items (17). Those with >10% missing were excluded from the analysis. The FI is a value between 0 and 1, derived by summing the scores of all present deficits and dividing by the total possible score. In this index, a higher score signifies more severe frailty. For analysis, the FI was used both as a continuous measure and as a binary variable, with frailty defined as FI ≥ 0.25 in accordance with prior studies (22, 23).

### 2.4 Assessment of covariates

Baseline covariates were collected using a structured questionnaire. Demographic variables included sex, age (continuous variable), ethnicity (Han, Zhuang, or other), marital status (married/cohabiting or other), annual income (<10,000, 10,000–30,000, or ≥30,000 RMB), and education level (below primary, primary, or high school and above). Smoking, alcohol consumption, and physical activity were among the lifestyle variables included. Based on past six-month self-report, current smokers were defined as ≥1 cigarette/day, and current drinkers as ≥1 time/week. Others were classified as non-smokers or non-drinkers. Physical activity was defined as engaging in continuous daily activity lasting ≥20 min during the past 6 months.

### 2.5 Statistical analysis

Means (SD) are presented for continuous variables and frequencies (%) for categorical variables. Groups were compared using the Mann–Whitney *U* test (continuous) and chi-square test (categorical).

Multivariable regression analyses were performed to investigate whether ePWV was associated with frailty. In analyses of baseline ePWV, logistic regression was used to calculate odds ratios (ORs) and 95% confidence intervals (CIs) for incident frailty (binary outcome) and generalized linear models were fitted to estimate regression coefficients (β) and 95% CIs for continuous FI. ePWV was treated as both a continuous variable and in quartiles, with trend tests performed across categories. Two adjusted models were built: Model 1 included age and sex; Model 2 further included all other baseline covariates. To explore the dose–response relationship, restricted cubic splines (RCS) were applied in Model 2 for baseline ePWV and FI. Nodes were set at the 10th, 50th, and 90th percentiles, and the 50th percentile was used as the reference value. The same analytical framework was applied for ΔePWV, with baseline ePWV additionally included in Model 2 to assess the independent effect of this longitudinal change over time.

Stratified analyses were conducted by age (≤70 or >70), sex, hypertension status, and antihypertensive treatment. Effect modification was tested using interaction terms and likelihood ratio tests. To ensure robustness, several sensitivity analyses were conducted. First, as 1,034 participants were lost to follow-up, Inverse probability weighting (IPW) was applied to address potential bias from non-random loss to follow-up (24). The inclusion probability for the final analytical cohort was estimated using a logistic regression model with baseline characteristics as predictors. The inverse of this probability was used as a weight in the primary multivariable regression (Model 2). Second, alternative cutoffs (P20/P80 and P30/P70) were used to reclassify ΔePWV groups to evaluate the consistency of results. Third, to assess whether the association between ΔePWV and frailty was independent of longitudinal blood pressure changes, we further adjusted for longitudinal changes in MBP (ΔMBP) in the multivariable model within Cohort 2. Fourth, to address potential conceptual overlap between ePWV and frailty, we conducted a sensitivity analysis excluding hypertension and cardiovascular disease from the 32 FI items. Finally, a sensitivity analysis was conducted using a composite outcome of incident frailty or death. Participants who either developed frailty or died before follow-up were classified as cases, while those who survived without frailty were classified as non-cases. Multivariable logistic regression was used to estimate the ORs for this composite outcome.

Data were analyzed with R (v4.2.1) and SPSS (v23.0). A two-sided *p*-value < 0.05 was considered statistically significant.

## 3 Results

### 3.1 Baseline characteristics of study participants

In the primary analysis (*n* = 2,016) used for baseline association analysis, the mean age was 68.6 years (61.1% female). During a mean follow-up of 7 years, 394 individuals (19.5%) developed frailty. Compared with non-frail individuals, those who developed frailty were older, had a higher proportion of females, lower education levels, and less baseline physical activity (Table 1). The mean baseline ePWV in the primary analysis was 11.4 (SD 1.6) m/s. Baseline characteristics of the secondary analysis (*n* = 1,137) are presented in Supplementary Table S2. Participants excluded from the initial baseline cohort (death, loss to follow-up, or missing data) were older, more likely male, higher educated, and less physically active at baseline than those included (Supplementary Table S3), suggesting potential non-random attrition. Among the primary analysis cohort, we further compared the 1,137 participants included in the secondary analysis with the 879 participants excluded due to missing follow-up blood pressure data. The two groups had no significant differences in baseline ePWV or FI (Supplementary Table S4). ePWV was moderately correlated with age (*r* = 0.697, *p* < 0.001), with a corresponding VIF of 1.94.

**Table 1:** Baseline characteristics by frailty status (*n* = 2016).

Characteristics	Overall (*N* = 2,016)	Non-frailty (*N* = 1,622)	Frailty (*N* = 394)	*P*
Sex, *n* (%)				0.001
Male	784 (38.89)	659 (40.63)	125 (31.73)	
Female	1,232 (61.11)	963 (59.37)	269 (68.27)	
Age, mean (SD)	68.61 (5.73)	68.12 (5.53)	70.62 (6.67)	<0.001
Ethnic, *n* (%)				0.167
Han	192 (9.52)	150 (9.25)	42 (10.66)	
Zhuang	1754 (87.01)	1,421 (87.61)	333 (84.52)	
Others	70 (3.47)	51 (3.14)	19 (4.82)	
Education, *n* (%)				<0.001
Less than primary school	925 (45.88)	703 (43.34)	222 (56.35)	
Primary school	693 (34.38)	577 (35.57)	116 (29.44)	
High school and above	398 (19.74)	342 (21.09)	56 (14.21)	
Marital status, *n* (%)				0.027
Married/cohabiting	1,427 (70.78)	1,166 (71.89)	261 (66.24)	
Others	589 (29.22)	456 (28.11)	133 (33.76)	
Annual income, *n* (%)				0.993
<10,000 RMB	762 (37.80)	614 (37.85)	148 (37.56)	
≥10,000 and <30,000	537 (26.64)	432 (26.63)	105 (26.65)	
≥30,000 RMB	717 (35.56)	576 (35.52)	141 (35.79)	
Cigarette smoking, *n* (%)				0.319
Non-smokers	1707 (84.67)	1,367 (84.28)	340 (86.29)	
Current smokers	309 (15.33)	255 (15.72)	54 (13.71)	
Alcohol drinking, *n* (%)				0.300
Nondrinkers	1,541 (76.44)	1,232 (75.96)	309 (78.43)	
Current drinkers	475 (23.56)	390 (24.04)	85 (21.57)	
Physical activities, *n* (%)				0.015
Yes	1924 (95.44)	1,557 (95.99)	367 (93.15)	
No	92 (4.56)	65 (4.01)	27 (6.85)	

Continuous variables were presented as mean ± SD. Categorical variables were presented as *n* (%).

SD, standard deviation; n, numbers of subjects; %, weighted percentage.

### 3.2 Association between baseline ePWV and frailty

The ePWV quartile cut-offs were Q1: <10.284 m/s, Q2: 10.284–11.220 m/s, Q3: 11.220–12.391 m/s, and Q4: ≥12.391 m/s. The number of frailty events in each quartile was 60 (Q1), 84 (Q2), 98 (Q3), and 152 (Q4), respectively. Baseline ePWV was positively correlated with frailty risk in multivariable analysis. Each 1 m/s increase in ePWV was associated with a 20.7% higher risk of frailty (OR = 1.207, 95% CI: 1.095–1.330, *p* < 0.001). The top ePWV quartile (Q4) showed over twice the frailty risk of the bottom quartile (Q1) (OR = 2.113, 95% CI: 1.360–3.282, *p* = 0.001), with a significant linear trend across quartiles (*P* for trend = 0.001) (Table 2). Similar associations emerged with FI treated as a continuous outcome. Per 1 m/s increase in ePWV, FI increased by 0.009 units (*β* = 0.009, 95% CI: 0.005–0.013, *p* < 0.001). Quartile analysis revealed a consistent dose–response relationship (vs. Q1: Q2, *β* = 0.013; Q3, *β* = 0.017; Q4, *β* = 0.036; all *p* < 0.05) (Table 3). RCS analysis indicated an approximately linear, positive dose–response relationship between baseline ePWV and FI (non-linear: *p* = 0.312, overall: *p* < 0.001) (Figure 2). Results remained robust in age- and sex-adjusted models.

**Table 2:** Logistic regression results for the association between baseline ePWV and incident frailty (*n* = 2016).

Baseline ePWV	Model 1 frailty		Model 2 frailty	
	Odds ratio (95% CIs)	*P*	Odds ratio (95% CIs)	*P*
ePWV	1.210 (1.100, 1.332)	<0.001	1.207 (1.095, 1.330)	<0.001
ePWV quartiles				
Q1	Reference		Reference	
Q2	1.333 (0.923, 1.925)	0.125	1.357 (0.938, 1.962)	0.105
Q3	1.458 (0.991, 2.147)	0.056	1.450 (0.982, 2.140)	0.062
Q4	2.146 (1.387, 3.320)	0.001	2.113 (1.360, 3.282)	0.001
	*P* for trend	0.001	*P* for trend	0.001

Association between baseline ePWV and incident frailty estimated by the logistic regression model. Model 1 were adjusted for sex, age. Model 2 were adjusted for sex, age, ethnicity, marital status, annual income, educational level, smoking, alcohol consumption, and physical activity. ePWV, estimated pulse wave velocity; Q, estimated pulse wave velocity quartiles.

**Table 3:** Generalized linear model results for the association between baseline ePWV and frailty index (*n* = 2016).

Baseline ePWV	Model 1 frailty index		Model 2 frailty index	
	*β* (95%CIs)	*P*	*β* (95%CIs)	*P*
ePWV	0.009 (0.006, 0.013)	<0.001	0.009 (0.005, 0.013)	<0.001
ePWV quartiles				
Q1	Reference		Reference	
Q2	0.013 (0.001, 0.024)	0.035	0.013 (0.001, 0.025)	0.027
Q3	0.017 (0.005, 0.030)	0.008	0.017 (0.005, 0.030)	0.008
Q4	0.036 (0.021, 0.052)	<0.001	0.036 (0.021, 0.052)	<0.001

Association between baseline ePWV and frailty index estimated by the generalized linear model. Model 1 were adjusted for sex, age. Model 2 were adjusted for sex, age, ethnicity, marital status, annual income, educational level, smoking, alcohol consumption, and physical activity.

ePWV, estimated pulse wave velocity; Q, estimated pulse wave velocity quartiles; β, regression coefficient; CI, confidence interval.

**Figure 2:**
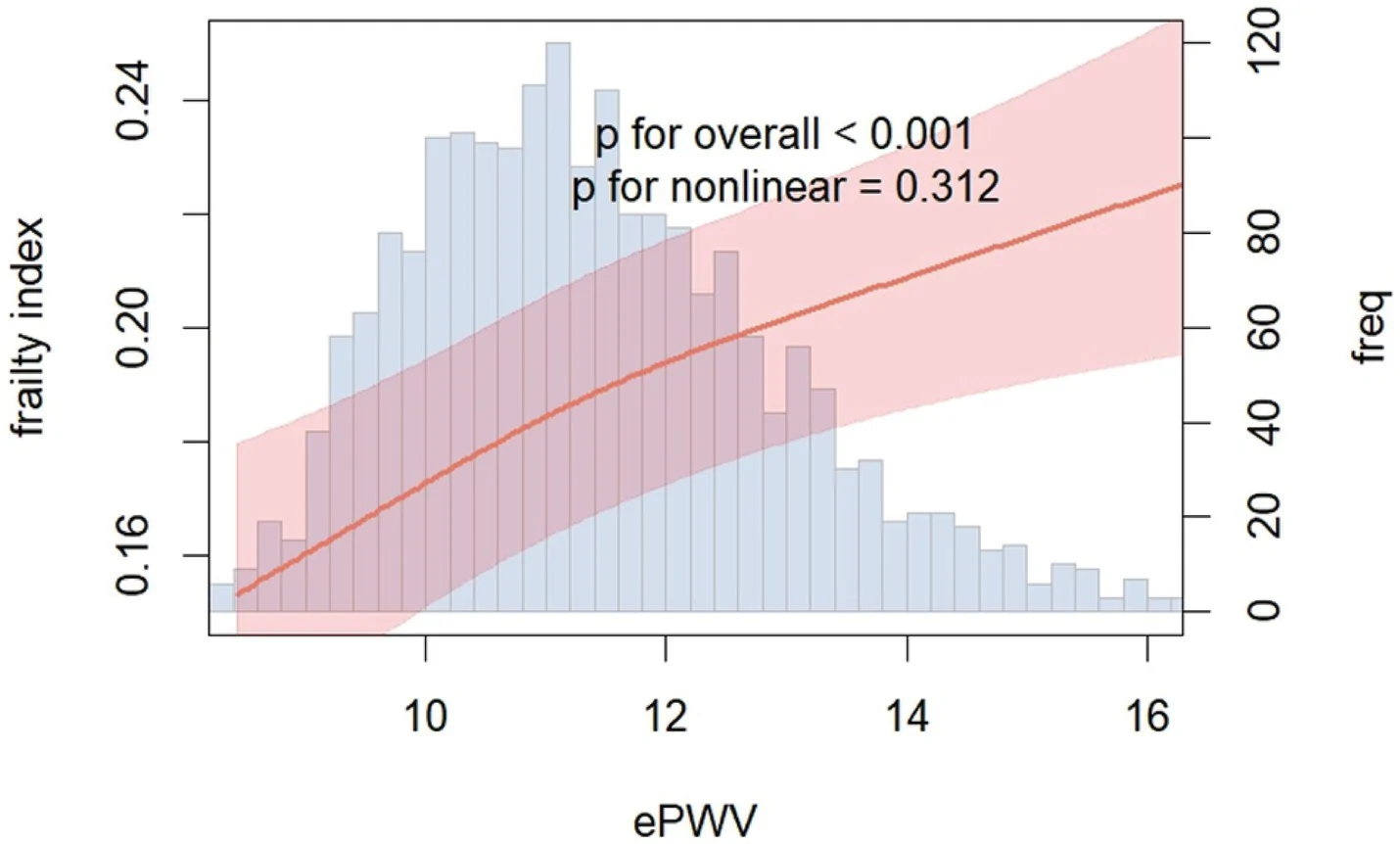
The restrictive cubic spline regression model (RCS) was used to investigate the dose–response relationship between baseline *ePWV* and frailty index (*FI*). Nodes were set at the 10th, 50th, and 90th percentiles, and the 50th percentile was used as the reference value. Models were adjusted for sex, age, ethnicity, marital status, annual income, educational level, smoking, alcohol consumption, and physical activity.

### 3.3 Association between longitudinal change in ePWV (ΔePWV) and frailty

An independent association was observed between rising ΔePWV and frailty. In the multivariable adjusted model, a 1 m/s increment in ΔePWV was associated with a 21.4% increased frailty risk (OR = 1.214, 95% CI: 1.028–1.434, *p* = 0.022). The rapid increase group exhibited significantly higher frailty risk than the decline/stable group (OR = 1.908, 95% CI: 1.126–3.233, *p* = 0.016) (Table 4). Consistent findings were observed for continuous FI. Per 1 m/s increase in ΔePWV, FI increased by 0.012 units (*β* = 0.012, 95% CI: 0.006–0.017, *p* < 0.001). Both the slow increase and rapid increase groups had significantly higher FI values than the decline/stable group, indicating a clear gradient of risk (slow increase: *β* = 0.016, 95CI: 0.003–0.029, *p* = 0.017; rapid increase: *β* = 0.037, 95CI: 0.021–0.054, *p* < 0.001) (Table 5).

**Table 4:** Logistic regression results for the association between longitudinal change in ePWV and incident frailty (*n* = 1,137).

Longitudinal change in ePWV	Event (%)	Model 1 frailty		Model 2 Frailty	
		Odds ratio (95% CIs)	*P*	Odds ratio (95% CIs)	*P*
ΔePWV		1.232 (1.045, 1.454)	0.013	1.214 (1.028, 1.434)	0.022
Changes in ePWV					
Decline/stable group	285 (25.07)	Reference		Reference	
Slow increase group	568 (49.96)	1.253 (0.807, 1.945)	0.314	1.257 (0.807, 1.956)	0.311
Rapid increase group	284 (24.98)	1.964 (1.166, 3.308)	0.011	1.908 (1.126, 3.233)	0.016

Association between longitudinal change in ePWV and incident frailty estimated by the logistic regression model. Model 1 were adjusted for sex, age. Model 2 were adjusted for sex, age, ethnicity, marital status, annual income, educational level, smoking, alcohol consumption, physical activity, and baseline ePWV.

Δ ePWV, longitudinal change in estimated pulse wave velocity; %, weighted percentage.

**Table 5:** Generalized linear model results for the association between longitudinal change in ePWV and frailty index (*n* = 1,137).

Longitudinal change in ePWV	Event (%)	Model 1 frailty index		Model 2 frailty index	
		*β* (95%CIs)	*P*	*β* (95%CIs)	*P*
ΔePWV		0.013 (0.008, 0.018)	<0.001	0.012 (0.006, 0.017)	<0.001
Changes in ePWV					
Decline/stable group	285 (25.07)	Reference		Reference	
Slow increase group	568 (49.96)	0.017 (0.004, 0.030)	0.011	0.016 (0.003, 0.029)	0.017
Rapid increase group	284 (24.98)	0.041 (0.024, 0.057)	<0.001	0.037 (0.021, 0.054)	<0.001

Association between longitudinal change in ePWV and frailty index estimated by the generalized linear model. Model 1 were adjusted for sex, age. Model 2 were adjusted for sex, age, ethnicity, marital status, annual income, educational level, smoking, alcohol consumption, physical activity, and baseline ePWV.

ΔePWV, longitudinal change in estimated pulse wave velocity; β, regression coefficient; CI, confidence interval; %, weighted percentage.

### 3.4 Stratified analyses

The consistency of the positive association between baseline ePWV and frailty was confirmed across subgroups based on age, sex, hypertension status, and antihypertensive treatment (Supplementary Figure S1a). No significant interactions were observed (all *P* for interaction > 0.05). The ΔePWV-frailty association remained consistent across all subgroups examined, with no evidence of effect modification (Supplementary Figure S1b).

### 3.5 Sensitivity analyses

Findings after applying IPW to account for attrition were similar to the primary analysis (Supplementary Table S5). In addition, associations were unchanged when alternative percentile cutoffs (P20/P80 and P30/P70) were used to classify ΔePWV (Supplementary Tables S6, S7). In the sensitivity analysis further adjusting for ΔMBP, ΔePWV as a continuous variable remained significantly associated with higher FI (*β* = 0.051, 95% CI: 0.008–0.094, *p* = 0.022), and the rapid increase group also remained significantly associated with higher FI (*β* = 0.028, 95% CI: 0.002–0.054, *p* = 0.035). However, the association with binary frailty was attenuated and no longer significant. Detailed results are presented in Supplementary Table S8. In a sensitivity analysis excluding hypertension and cardiovascular disease from the FI, the associations of both baseline ePWV (*β* = 0.003, 95% CI: −0.001–0.006, *p* = 0.115) and ΔePWV (*β* = 0.003, 95% CI: −0.002–0.009, *p* = 0.195) with the recalculated FI were attenuated and no longer statistically significant (Supplementary Table S9). In the sensitivity analysis using a composite outcome of incident frailty or death (*n* = 2,773), higher baseline ePWV was significantly associated with an increased risk of the composite outcome (OR = 1.233, 95% CI: 1.151–1.322, *p* < 0.001), consistent with the primary findings (Supplementary Table S10).

## 4 Discussion

This study demonstrates that ePWV was significantly associated with incident frailty. Both higher baseline ePWV and greater increases over time were associated with higher frailty risk. Baseline ePWV showed a clear linear association with the FI, and participants with a more rapid increase in ePWV experienced a graded elevation in frailty risk. These findings suggest that frailty risk in older adults is linked not only to the absolute level of baseline ePWV but also to increases in ePWV across the 7-year follow-up.

Our findings are consistent with previous studies reporting associations between arterial stiffness and frailty (12, 25). However, as noted in a systematic review (10), most existing evidence is derived from cross-sectional studies, which limits the ability to infer temporal sequence or causality. Importantly, arterial stiffness is widely recognized as a progressive process (15). Although several longitudinal studies have investigated the associations of cfPWV and baPWV with frailty outcomes (13, 14), prospective evidence on ePWV, a more readily accessible surrogate marker of arterial stiffness, and its longitudinal change in relation to incident frailty remains scarce. The significant ΔePWV-frailty association persisted after adjustment for baseline ePWV, suggesting that increases in ePWV over time may carry additional prognostic value. These findings support the notion that ePWV may reflect an ongoing vascular risk process, warranting further evaluation of its dynamic monitoring value. Given that ePWV is simple and non-invasive, it may have potential as a practical indicator for frailty risk stratification, although further validation is required.

Our study also provides insight into the pattern of association. We identified a pattern that differs from previous cross-sectional report. In contrast to a previous cross-sectional study suggesting a potential nonlinear relationship among patients with cardiometabolic disease (12), we observed a linear dose–response relationship in a community-based older population. Several factors may explain this difference. The prospective design reduces the likelihood of reverse causation. In addition, analyzing frailty as a measure captures its gradual progression more effectively than a dichotomous classification. Moreover, a community-based sample may better reflect the natural aging process. Together, these features may explain the observed linear association, suggesting that frailty risk increases continuously across the full range of ePWV. This aligns with evidence that arterial stiffness broadly and continuously influences diverse health outcomes including hypertension, cardiovascular disease, and all-cause mortality (26, 27).

Several mechanisms may underlie this relationship. Arterial stiffness and frailty may share common upstream processes related to chronic low-grade inflammation associated with aging (28). Persistent inflammatory stress can directly damage vessels, muscle, neural and metabolic function to induce multisystem injury (29, 30), and can also increase arterial stiffness (31). Elevated arterial stiffness impairs the pressure-buffering capacity of large arteries, allowing excessive pulsatile energy to be transmitted to the microcirculation of high-flow organs across the body (32). This pulsatile overload is hypothesized to promote frailty through organ-specific microvascular injury. In skeletal muscle, this heightened pulsatile load may impair capillary perfusion and endothelial function, reducing nutrient and oxygen delivery and contributing to sarcopenia and declining physical function (33). In the cerebral microcirculation, excessive pulsatility can disrupt microvascular integrity and promote white matter hyperintensities, which are linked to cognitive decline (34). In the kidney, increased pulsatile pressure at the glomerular capillary level may induce glomerular hypertension and endothelial injury, accelerating the decline in renal function (35). Collectively, pulsatile overload secondary to elevated arterial stiffness, together with inflammatory injury, jointly drive the multisystem functional decline characteristic of frailty.

This study demonstrates several strengths. First, it employed a prospective cohort design, which provides better evidence than cross-sectional studies for establishing temporal sequence. Second, we evaluated both baseline ePWV and its longitudinal change with adjustment for baseline ePWV, providing a more comprehensive assessment of dynamic changes in this marker. Third, our findings were robust across different outcome definitions and analytical approaches. Nonetheless, limitations should be considered. First, ePWV was calculated using an established formula rather than being measured directly as cfPWV, though it has comparable predictive value to measured cfPWV (9, 36). Caution is needed when interpreting ΔePWV. Since ePWV is calculated from age and mean blood pressure, its longitudinal change may not solely reflect true arterial stiffening. Second, the apparent linear dose–response relationship between ePWV and frailty should be interpreted with caution, as it may partly reflect the age–frailty association rather than a direct biological linear relationship with measured arterial stiffness. Third, ePWV was assessed at only two time points, limiting evaluation of more detailed trajectories. In addition, substantial sample attrition due to death, loss to follow-up, and missing follow-up data may have introduced selection bias and limit the generalizability of the findings. Although sensitivity analyses using a composite outcome and using IPW yielded consistent results, the possibility of residual selection bias cannot be entirely excluded. Fourth, despite extensive adjustment for confounders, residual bias due to unmeasured confounding (e.g., kidney function, polypharmacy, and diet) cannot be ruled out. Additionally, physical activity was assessed using a simple binary measure, which may not fully capture its intensity or duration. Fifth, sensitivity analyses excluding cardiovascular-related FI items substantially attenuated the associations of ePWV and ΔePWV with frailty, rendering the results non-significant. These findings indicate that the observed association may be partially driven by cardiovascular components within the FI, warranting cautious interpretation of our main results. Sixth, the geographic confinement to specific Chinese regions may limit generalizability. Finally, given the observational approach, these findings represent associations only; interventional studies remain necessary to establish causality.

In conclusion, higher baseline ePWV and greater increases in ePWV over follow-up were associated with increased frailty risk in older adults, although part of this association may be related to the cardiovascular components included within the FI. These findings highlight the potential value of ePWV as a practical indicator for identifying individuals at elevated risk. Future research is needed to corroborate these results in diverse populations, clarify clinically meaningful thresholds, and explore whether interventions targeting arterial stiffness can reduce frailty risk.

## Data Availability

The datasets presented in this article are not readily available because the datasets analyzed during the current study is not publicly available due to privacy and confidentiality concerns. However, they are available from the corresponding author, Xiaobo Yang, on reasonable request. Requests to access the datasets should be directed to Xiaobo Yang, yangx@gxmu.edu.cn.
