# Supplementary material for: Baseline estimated pulse wave velocity (ePWV) and its longitudinal change are associated with incident frailty in older Chinese adults: a prospective cohort study
Source: Front Public Health. 2026 Oct 28;14:1926256. doi: 10.3389/fpubh.2026.1926256 (PMC13751520; doi:10.3389/fpubh.2026.1926256)
Supplement: Supplementary file 1 [file Table_1.DOCX]

| Table S1 The relevant variable used to construct the frailty index (n=4484) | | |
| --- | --- | --- |
| Variable name | Specific items | Coding |
| Disease (8 items) | hypertension | no=0, yes=1 |
|  | diabetes | no=0, yes=1 |
|  | cardiovascular disease | no=0, yes=1 |
|  | cancer | no=0, yes=1 |
|  | osteoarthropathy | no=0, yes=1 |
|  | osteoporosis | no=0, yes=1 |
|  | COPD | no=0, yes=1 |
|  | rheumatism | no=0, yes=1 |
|  |  |  |
| Activities of daily living (14 items) | bathing |  |
|  | dressing |  |
|  | toileting |  |
|  | transferring |  |
|  | eating |  |
|  | continence | without difficulties = 0, |
|  | telephone calls | some difficulties = 0.33, |
|  | shopping | considerable difficulties = 0.67, |
|  | transportation | impossible = 1 |
|  | handing medications |  |
|  | managing finances |  |
|  | doing the laundry |  |
|  | preparing meals |  |
|  | housekeeping |  |
|  |  |  |
| General health (2 items) | general self-rated health | excellent=0.00, good=0.33 |
|  |  | fair=0.67, Poor=1.00 |
|  | life satisfaction | very satisfied = 0.00, satisfied = 0.33 |
|  |  | fair = 0.67, unsatisfactory = 1.00 |
|  |  |  |
| Signs and Symptoms (8 items) | sleep deficits | no=0.00, yes=1.00 |
|  | pain | no=0.00, yes=1.00 |
|  | hospitalization (last year) | no=0.00, yes=1.00 |
|  | hearing | normal = 0.00, slightly clear = 0.33; |
|  |  | relatively clear = 0.67, severe drop = 1.00 |
|  | heart rate | <60 or >100=1.00; ≥60 and ≤100=0.00 |
|  | body mass index (kg/m²) | <18.5 or ≥28.0=1.00; ≥24.0 and <28.0=0.50; ≥18.5 and <24.0=0.00 |
|  | waist-to-hip ratio | male:≥0.95 =1.00; ≥0.90 and <0.95=0.50;<0.90 =0.00 female:≥0.90 =1.00; ≥0.85 and <0.90=0.50; <0.85 =0.00 |
|  | grip strength | lowest 20% (by gender, BMI) |

| Table S2 Baseline characteristics by frailty status (n=1137) | | | | |
| --- | --- | --- | --- | --- |
|  | Overall (N=1137) | non-frailty (N=923) | frailty (N=214) | *P* |
| Sex, n(%) |  |  |  | 0.001 |
| Male | 445 (39.14) | 382 (41.39) | 63 (29.44) |  |
| Female | 692 (60.86) | 541 (58.61) | 151 (70.56) |  |
| Age, mean (SD) | 68.42 (5.47) | 67.96 (5.32) | 70.42 (5.65) |  |
| Ethnic, n (%) |  |  |  | 0.909 |
| Han | 131 (11.52) | 105 (11.38) | 26 (12.15) |  |
| Zhuang | 967 (85.05) | 787 (85.27) | 180 (84.11) |  |
| Others | 39 (3.43) | 31 (3.36) | 8 (3.74) |  |
| Education, n (%) |  |  |  | ＜0.001 |
| Less than primary school | 519 (45.65) | 395 (42.80) | 124 (57.94) |  |
| Primary school | 401 (35.27) | 340 (36.84) | 61 (28.50) |  |
| High school and above | 217 (19.09) | 188 (20.36) | 29 (13.55) |  |
| Marital status, n (%) |  |  |  | 0.015 |
| Married/cohabiting | 820 (72.12) | 680 (73.67) | 140 (65.42) |  |
| Others | 317 (27.88) | 243 (26.33) | 74 (34.58) |  |
| Annual income, n (%) |  |  |  | 0.739 |
| <10,000 RMB | 417 (36.68) | 340 (36.84) | 77 (35.98) |  |
| ≥10,000 and <30,000 | 304 (26.74) | 250 (27.09) | 54 (25.23) |  |
| ≥30,000 RMB | 416 (36.58) | 333 (36.07) | 83 (38.79) |  |
| Cigarette smoking, n (%) |  |  |  | 0.347 |
| Nonsmokers | 959 (84.34) | 774 (83.86) | 185 (86.45) |  |
| Current smokers | 178 (15.66) | 149 (16.14) | 29 (13.55) |  |
| Alcohol drinking, n (%) |  |  |  | 0.167 |
| Nondrinkers | 873 (76.78) | 701 (75.95) | 172 (80.37) |  |
| Current drinkers | 264 (23.22) | 222 (24.05) | 42 (19.63) |  |
| Physical activities, n (%) |  |  |  | 0.299 |
| Yes | 1088 (95.69) | 886 (95.99) | 202 (94.39) |  |
| No | 49 (4.31) | 37 (4.01) | 12 (5.61) |  |

Continuous variables were presented as mean ± SD. Categorical variables were presented as n (%).

Abbreviation: SD: standard deviation; n: numbers of subjects; %: weighted percentage.

| Table S3 Baseline characteristics of included and excluded participants | | | |
| --- | --- | --- | --- |
|  | Included (N=2016) | Excluded (N=2113) | *P* |
| Sex, n(%) |  |  | 0.004 |
| Male | 784 (38.89) | 914 (43.26) |  |
| Female | 1232 (61.11) | 1199 (56.74) |  |
| Age, mean (SD) | 68.61 (5.73) | 70.68 (7.58) | ＜0.001 |
| Ethnic, n (%) |  |  |  |
| Han | 192 (9.52) | 210 (9.94) | 0.011 |
| Zhuang | 1754 (87.00)) | 1790 (84.71) |  |
| Others | 70 (3.48) | 113 (5.35) |  |
| Education, n (%) |  |  | 0.029 |
| Less than primary school | 925 (45.88) | 926 (43.82) |  |
| Primary school | 693 (34.38) | 698 (33.03) |  |
| High school and above | 398 (19.74) | 489 (23.15) |  |
| Marital status, n (%) |  |  | 0.076 |
| Married/cohabiting | 1427 (70.78) | 1442 (68.24) |  |
| Others | 589 (29.22) | 671 (31.76) |  |
| Annual income, n (%) |  |  | 0.813 |
| <10,000 RMB | 762 (37.80) | 781 (36.96) |  |
| ≥10,000 and <30,000 | 537 (26.63) | 562 (26.60) |  |
| ≥30,000 RMB | 717 (35.57) | 770 (36.44) |  |
| Cigarette smoking, n (%) |  |  | 0.070 |
| Nonsmokers | 1707 (84.67) | 1745 (82.58) |  |
| Current smokers | 309 (15.33) | 368 (17.42) |  |
| Alcohol drinking, n (%) |  |  | 0.270 |
| Nondrinkers | 1541 (76.44) | 1584 (74.96) |  |
| Current drinkers | 475 (23.46) | 529 (25.04) |  |
| Physical activities, n (%) |  |  | ＜0.001 |
| Yes | 1924 (95.44) | 1892 (89.54) |  |
| NO | 92 (4.56) | 221 (10.46) |  |

Continuous variables were presented as mean ± SD. Categorical variables were presented as n (%). Abbreviation: SD: standard deviation; n: numbers of subjects; %: weighted percentage.

| Table S4. Baseline characteristics of participants with and without available follow-up blood pressure data. | | | |
| --- | --- | --- | --- |
| Characteristics | Included (N=1137) | Excluded (N=879) | *P* |
| Sex, n (%) |  |  | 0.794 |
| Male | 445 (39.14) | 339 (38.57) |  |
| Female | 692 (60.86) | 540 (61.43) |  |
| Age, mean (SD) | 68.42 (5.47) | 68.86 (6.04) | 0.262 |
| Ethnic, n (%) |  |  | 0.002 |
| Han | 131 (11.52) | 61 (6.94) |  |
| Zhuang | 967 (85.05) | 787 (89.53) |  |
| Others | 39 (3.43) | 31 (3.53) |  |
| Education, n (%) |  |  | 0.547 |
| Less than primary school | 519 (45.65) | 406 (46.19) |  |
| Primary school | 401 (35.27) | 292 (33.22) |  |
| High school and above | 217 (19.08) | 181 (20.59) |  |
| Marital status, n (%) |  |  | 0.134 |
| Married/cohabiting | 820 (72.12) | 607 (69.06) |  |
| Others | 317 (27.88) | 272 (30.94) |  |
| Annual income, n (%) |  |  | 0.439 |
| <10,000 RMB | 417 (36.68) | 345 (39.25) |  |
| ≥10,000 and <30,000 | 304 (26.73) | 233 (26.51) |  |
| ≥30,000 RMB | 416 (36.59) | 301 (34.24) |  |
| Cigarette smoking, n (%) |  |  | 0.642 |
| Nonsmokers | 959 (84.34) | 748 (85.10) |  |
| Current smokers | 178 (15.66) | 131 (14.90) |  |
| Alcohol drinking, n (%) |  |  | 0.680 |
| Nondrinkers | 873 (76.78) | 668 (76.00) |  |
| Current drinkers | 264 (23.22) | 211 (24.00) |  |
| Physical activities, n (%) |  |  | 0.534 |
| Yes | 1088 (95.69) | 836 (95.11) |  |
| NO | 49 (4.31) | 43 (4.89) |  |
| Baseline ePWV | 11.38 | 11.50 | 0.201 |
| Baseline frailty index | 0.12 | 0.12 | 0.695 |

Continuous variables were presented as mean ± SD. Categorical variables were presented as n (%). Abbreviation: SD: standard deviation; n: numbers of subjects; %: weighted percentage.

| Table S5 The association between baseline ePWV and its longitudinal change with incident frailty using inverse probability weighting. | | | | |
| --- | --- | --- | --- | --- |
| ePWV | frailty | | frailty index | |
|  | Odds ratio (95% CIs) | *P* | β (95%CI) | *P* |
| baseline ePWV | 1.208 (1.098, 1.329) | ＜0.001 | 0.009 (0.006, 0.013) | ＜0.001 |
| ΔePWV | 1.218 (1.084, 1.368) | 0.001 | 0.014 (0.009, 0.018) | ＜0.001 |

Association between baseline ePWV and its longitudinal change with incident frailty using inverse probability weighting. Model were adjusted for sex, age, ethnicity, marital status, annual income, educational level, smoking, alcohol consumption, physical activity, or baseline ePWV.

Abbreviation: ePWV: estimated pulse wave velocity; ΔePWV: longitudinal change in estimated pulse wave velocity; β: regression coefficient; CI: confidence interval.

| Table S6 The association between longitudinal change in ePWV and incident frailty by redefining categorical thresholds at the 20th and 80th percentiles. | | | | |
| --- | --- | --- | --- | --- |
| ePWV longitudinal change | frailty | | frailty index | |
|  | Odds ratio(95% CIs) | *P* | β（95%CI） | *P* |
| decline/stable group | Reference |  | Reference |  |
| slow increase group | 1.842 (1.122, 3.022) | 0.016 | 0.023 (0.009, 0.038) | 0.001 |
| rapid increase group | 2.457 (1.328, 4.547) | 0.004 | 0.042 (0.024, 0.061) | 0.001 |

Association between longitudinal change in ePWV and incident frailty by redefining categorical thresholds at the 20th and 80th percentiles. Model were adjusted for sex, age, ethnicity, marital status, annual income, educational level, smoking, alcohol consumption, physical activity, and baseline ePWV.

Abbreviation: β: regression coefficient; CI: confidence interval.

| Table S7 The association between longitudinal change in ePWV and incident frailty by redefining categorical thresholds at the 30th and 70th percentiles. | | | | |
| --- | --- | --- | --- | --- |
| ePWV longitudinal change | frailty | | frailty index | |
|  | Odds ratio (95% CIs) | *P* | β (95%CI) | *P* |
| decline/stable group | Reference |  | Reference |  |
| slow increase group | 1.190 (0.775, 1.827) | 0.425 | 0.011 (-0.001, 0.024) | 0.082 |
| rapid increase group | 1.886 (1.168, 3.046) | 0.009 | 0.038(0.023, 0.052) | 0.001 |

Association between longitudinal change in ePWV and incident frailty by redefining categorical thresholds at the 30th and 70th percentiles. Model were adjusted for sex, age, ethnicity, marital status, annual income, educational level, smoking, alcohol consumption, physical activity, and baseline ePWV.

Abbreviation: β: regression coefficient; CI: confidence interval.

| Table S8. Sensitivity analysis adjusting for longitudinal changes in mean blood pressure (ΔMBP) for the association between ΔePWV and frailty | | | | | | |
| --- | --- | --- | --- | --- | --- | --- |
| Outcome | longitudinal change in ePWV | Without adjustment |  | With adjustment | |  |
| Frailty |  | OR (95% CI) | *P* | OR (95% CI) | *P* | |
|  | ΔePWV | 1.214 (1.028–1.434) | 0.022 | 2.389 (0.623–9.163) | | 0.204 |
|  | decline/stable group | Reference |  | Reference | |  |
|  | slow increase group | 1.257 (0.807, 1.956) | 0.311 | 1.189 (0.685, 2.062) | | 0.539 |
|  | rapid increase group | 1.908 (1.126–3.233) | 0.016 | 1.713 (0.750–3.912) | | 0.201 |
| frailty index |  | β (95% CI) | *P* | β (95% CI) | | *P* |
|  | ΔePWV | 0.012 (0.006–0.017) | <0.001 | 0.051 (0.008–0.094) | | 0.022 |
|  | decline/stable group | Reference |  | Reference | |  |
|  | slow increase group | 0.016 (0.003–0.029) | 0.017 | 0.011 (−0.005–0.028) | | 0.184 |
|  | rapid increase group | 0.037 (0.021–0.054) | <0.001 | 0.028 (0.002–0.054) | | 0.035 |

All models were adjusted for sex, age, ethnicity, marital status, annual income, educational level, smoking, alcohol consumption, physical activity, and baseline ePWV. was defined as follow‑up MBP minus baseline MBP. CI, confidence interval; OR, odds ratio.

Abbreviation: ΔMBP, longitudinal changes in mean blood pressure; CI, confidence interval; OR, odds ratio

| Table S9. Sensitivity analysis excluding cardiovascular items from the frailty index. | | | | |
| --- | --- | --- | --- | --- |
| Exposure | Original frailty index | | Excluding cardiovascular items | |
|  | β (95% CI) | *P* | β (95% CI) | *P* |
| Baseline ePWV | 0.009 (0.005, 0.013) | ＜0.001 | 0.003 (-0.001, 0.006) | 0.115 |
| ΔePWV | 0.012 (0.006, 0.017) | ＜0.001 | 0.003 (-0.002, 0.009) | 0.195 |

Model were adjusted for sex, age, ethnicity, marital status, annual income, educational level, smoking, alcohol consumption, physical activity, or baseline ePWV.

Abbreviation: ePWV: estimated pulse wave velocity; ΔePWV: longitudinal change in estimated pulse wave velocity; β: regression coefficient; CI: confidence interval.

| Table S10 Sensitivity analysis using a composite outcome of incident frailty or death. | | |
| --- | --- | --- |
| Variable | OR (95% CIs) | *P* |
| ePWV | 1.233 (1.151, 1.322) | < 0.001 |

This analysis included 2,773 participants with ascertainable outcome status from the initial non-frail baseline cohort. The composite outcome was defined as the occurrence of death or incident frailty. Model were adjusted for sex, age, ethnicity, marital status, annual income, educational level, smoking, alcohol consumption, and physical activity.

Abbreviation: ePWV: estimated pulse wave velocity; OR, odds ratio.

Figures:

**Figure S1. Stratified analysis of the ePWV-frailty association by age (≤70 vs >70), sex (male vs female), hypertension status (NO vs YES), and antihypertensive treatment (NO vs YES).** (a) Association between baseline ePWV and frailty across the specified subgroups. (b) Association between dynamic change in ePWV and frailty across the specified subgroups.
